# High-Throughput Chemical Screen Identifies a 2,5-Disubstituted Pyridine as an Inhibitor of Candida albicans Erg11

**DOI:** 10.1128/msphere.00075-22

**Published:** 2022-05-09

**Authors:** Antonia C. Du Bois, Alice Xue, Chester Pham, Nicole M. Revie, Kirsten J. Meyer, Yoko Yashiroda, Charles Boone, Justin R. Nodwell, Peter Stogios, Alexei Savchenko, Nicole Robbins, Kali R. Iyer, Leah E. Cowen

**Affiliations:** a Department of Molecular Genetics, University of Toronto, Toronto, Ontario, Canada; b Department of Chemical Engineering and Applied Chemistry, University of Toronto, Toronto, Ontario, Canada; c Department of Biochemistry, University of Toronto, Toronto, Ontario, Canada; d RIKEN Center for Sustainable Resource Science, Wako, Saitama, Japan; e Donnelly Centre for Cellular and Biomedical Research, University of Toronto, Toronto, Ontario, Canada; f Department of Microbiology, Immunology and Infectious Diseases, Cumming School of Medicine, University of Calgary, Calgary, Alberta, Canada; University of Georgia

**Keywords:** 2, 5-disubstituted pyridine, *Candida albicans*, Erg11, azole, chemogenomics, computational modeling, ergosterol, fungal pathogen

## Abstract

Fungal infections contribute to over 1.5 million deaths annually, with Candida albicans representing one of the most concerning human fungal pathogens. While normally commensal in nature, compromise of host immunity can result in C. albicans disseminating into the human bloodstream, causing infections with mortality rates of up to 40%. A contributing factor to this high mortality rate is the limited arsenal of antifungals approved to treat systemic infections. The most widely used antifungal class, the azoles, inhibits ergosterol biosynthesis by targeting Erg11. The rise of drug resistance among C. albicans clinical isolates, particularly against the azoles, has escalated the need to explore novel antifungal strategies. To address this challenge, we screened a 9,600-compound subset of the University of Tokyo Core Chemical Library to identify molecules with novel antifungal activity against C. albicans. The most potent hit molecule was CpdLC-6888, a 2,5-disubstituted pyridine compound, which inhibited growth of C. albicans and closely-related species. Chemical-genetic, biochemical, and modeling analyses suggest that CpdLC-6888 inhibits Erg11 in a manner similar to the azoles despite lacking the canonical five-membered nitrogen-containing azole ring. This work characterizes the antifungal activity of a 2,5-disubstituted pyridine against C. albicans, supporting the mining of existing chemical collections to identify compounds with novel antifungal activity.

**IMPORTANCE** Pathogenic fungi represent a serious but underacknowledged threat to human health. The treatment and management of these infections relies heavily on the use of azole antifungals, a class of molecules that contain a five-membered nitrogen-containing ring and inhibit the biosynthesis of the key membrane sterol ergosterol. By employing a high-throughput chemical screen, we identified a 2,5-disubstituted pyridine, termed CpdLC-6888, as possessing antifungal activity against the prominent human fungal pathogen Candida albicans. Upon further investigation, we determined this molecule exhibits azole-like activity despite being structurally divergent. Specifically, transcriptional repression of the azole target gene *ERG11* resulted in hypersensitivity to CpdLC-6888, and treatment of C. albicans with this molecule blocked the production of the key membrane sterol ergosterol. Therefore, this work describes a chemical scaffold with novel antifungal activity against a prevalent and threatening fungal pathogen affecting human health, expanding the repertoire of compounds that can inhibit this useful antifungal drug target.

## OBSERVATION

Fungal pathogens are a significant threat to human health, infecting over a billion people globally and resulting in 1.5 million deaths annually (1). Candida albicans is a natural constituent of the human microbiota; however, in immunocompromised individuals it can become pathogenic and cause serious systemic infections with resulting mortality rates of up to 40% (1–3). Currently there are only three major classes of antifungals available to treat these systemic infections: azoles, echinocandins, and polyenes. The azoles are a class of five-membered, nitrogen-containing heterocyclic compounds that inhibit the biosynthesis of the essential cell membrane component, ergosterol, by targeting the lanosterol demethylase enzyme encoded by *ERG11.* This results in the production of aberrant sterols, which exert membrane stress and ultimately halt yeast growth (4). Azoles are the most widely deployed antifungal drug class, as they are broad spectrum, have a good safety profile, and are orally bioavailable (4). However, their extensive deployment, paired with fungistatic mode of action, has spurred the emergence of drug-resistant isolates, which threatens their utility (4). Thus, it is crucial to explore and develop new antifungal treatment strategies.

### Chemical screen identifies CpdLC-6888 as a compound with activity against C. albicans.

**FIG 1:**
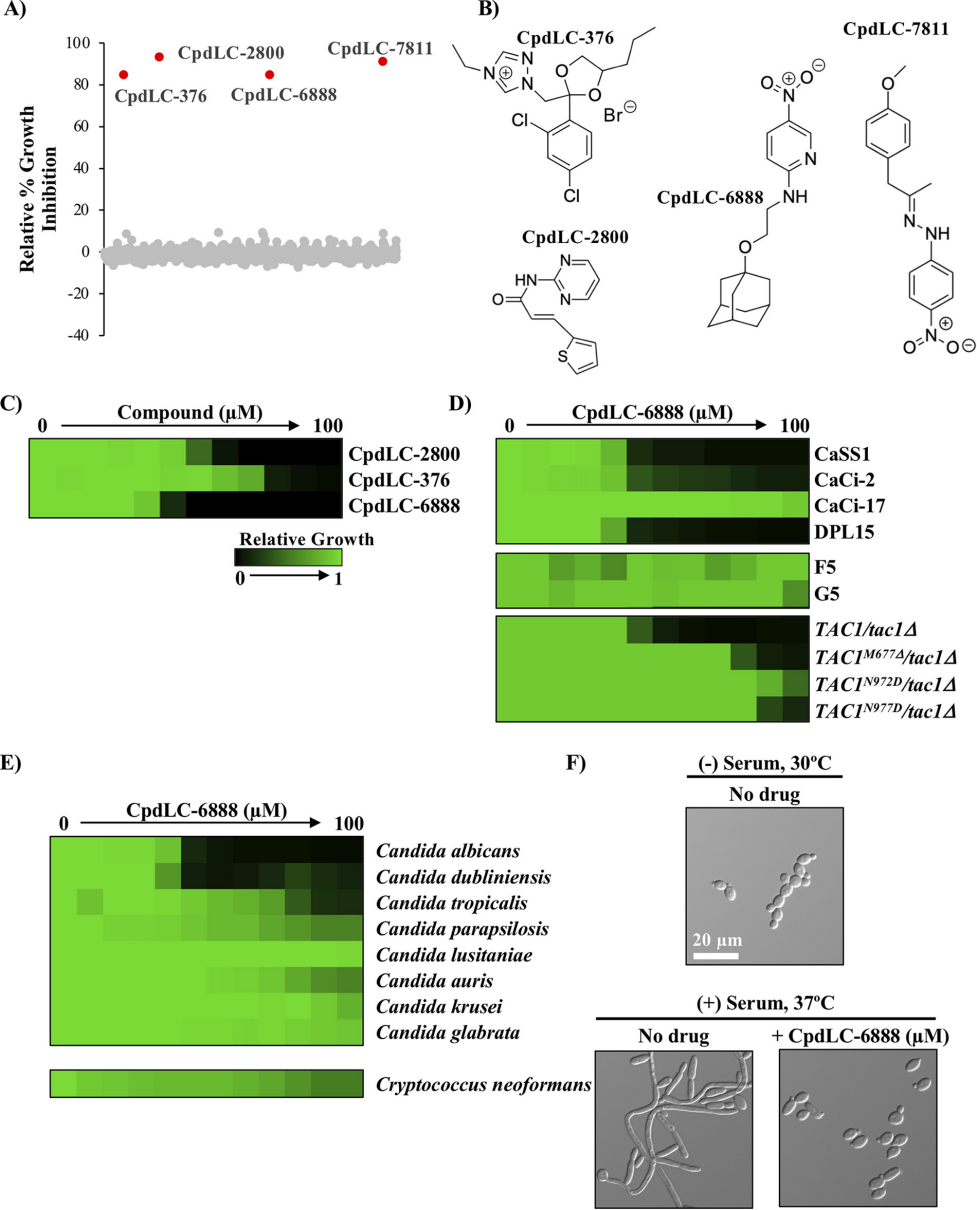
CpdLC-6888 exhibits potent bioactivity against C. albicans and closely related species. (A) Relative growth inhibition of C. albicans CaSS1 for each compound screened at 10 μM in YPD at 30°C for 24 h. Hit compounds, shown in red, were defined as conferring >80% growth inhibition. (B) Chemical structures of the four hit compounds. Note for CpdLC-2800, the E isomer (as shown) was the compound screened; however, the E/Z racemic mixture was used for validation in panel C. (C) Potency of hit compounds was assessed using a dose-response assay. C. albicans CaSS1 was grown in a 2-fold dilution series of the indicated compound in YPD at 30°C for 24 h. Growth was assessed by measuring optical density at 600 nm (OD_600_). Values were normalized relative to the respective no-drug control well (see color bar). (D) Dose-response assays with CpdLC-6888 performed as described for panel C for the C. albicans screening strain, CaSS1, the azole-tolerant and -resistant C. albicans clinical isolates, CaCi-2 and CaCi-17, the clinical isolates F5 and G5, and the echinocandin-resistant isolate DPL15. The *MRR1* allele in F5 contains a mutation that leads to a P683S substitution, and the *MRR1* allele in G5 contains a mutation that leads to a G977V substitution. Laboratory-derived strains harboring mutations in the transcriptional activator gene *TAC1* were also assessed. See color bar in panel C. (E) CpdLC-6888 dose-response assay was performed as described for panel C with a panel of fungal pathogens. Growth was measured after 24 h for *Candida* species and after 72 h for C. neoformans. Strains are ordered by phylogenetic relatedness to C. albicans. See color bar in panel C. (F) The effect of CpdLC-6888 on filamentation of C. albicans SN95 was assessed after incubation of cells in YPD medium with 2.5 μM CpdLC-6888 for 4 h at 30°C or 37°C, in the presence or absence of serum, as indicated. Images were taken by differential interference contrast microscopy at 40X magnification.

To identify compounds with novel antifungal activity against C. albicans, a high-throughput screen of a 9,600-chemical subset of the University of Tokyo’s “Core Library” was performed. This subset includes molecules that exhibit more drug-like features based on structural and physical properties while also enriching for structural diversity. Four distinct hit compounds were identified that inhibited growth of C. albicans by more than 80% relative to drug-free controls (Fig. 1A and B), three of which (CpdLC-2800, CpdLC-376, and CpdLC-6888) were procurable from an independent supplier. The potency of each compound against C. albicans was assessed via a standard dose-response assay in which growth of cells was measured in a 2-fold dilution series of each compound (Text S1) (5). The minimum inhibitory concentration (MIC) that reduced growth by 90% (MIC_90_) was calculated, with CpdLC-6888 emerging as the most potent compound exhibiting a MIC_90_ value of 3.13 μM in yeast extract-peptone-dextrose (YPD) (Fig. 1C) and a MIC_70_ value of 1.56 μM in RPMI (data not shown), making it the focus of further investigation.

10.1128/msphere.00075-22.4 TEXT S1 Supplemental methods used in this study. Download Text S1, DOCX file, 0.04 MB. Copyright © 2022 Du Bois et al. 2022 Du Bois et al. https://creativecommons.org/licenses/by/4.0/ This content is distributed under the terms of the Creative Commons Attribution 4.0 International license.

Next, the activity of CpdLC-6888 against diverse C. albicans clinical isolates was evaluated. CaCi-2 and CaCi-17 are isolates obtained from an HIV patient undergoing azole treatment, with CaCi-2 representing an azole-tolerant strain collected early during treatment and CaCi-17 an azole-resistant strain isolated later in treatment (6, 7). Specifically, CaCi-17 overexpresses a mutant form of Erg11 that contains a homozygous R467K substitution as well as overexpresses ABC efflux pumps due to a heterozygous substitution in the transcriptional activator Tac1 (6, 7). F5 and G5 represent clinical isolates that overexpress the major facilitator transporter Mdr1 due to substitutions in the transcriptional activator Mrr1 (8, 9). In contrast, DPL15 is a strain harboring mutations in the echinocandin drug target gene *FKS1* (10) (Fig. 1D). CpdLC-6888 showed potency against DPL15 equivalent to that of the laboratory screening strain CaSS1. In contrast, CaCi-2 and CaCi-17 demonstrated increasing resistance to CpdLC-6888, and F5 and G5 were resistant to all concentrations tested (Fig. 1D). Notably, the resistance observed with CaCi-17 was at least in part due to increased expression of ABC transporters, as laboratory-derived strains that specifically harbored substitutions in Tac1 were also resistant to CpdLC-6888 (11) (Fig. 1D). To further assess CpdLC-6888's spectrum of activity, dose-response assays were conducted against a panel of pathogenic yeasts, including non-*albicans Candida* species and the basidiomycete Cryptococcus neoformans (Fig. 1E). CpdLC-6888 exhibited potent activity against C. albicans and its closest relative, Candida dubliniensis, moderate activity against the next closest relative, Candida tropicalis, and minimal to no activity against the more divergent species (Fig. 1E). Finally, to further characterize the bioactivity of CpdLC-6888, we assessed its ability to block the C. albicans morphogenetic transition between yeast and filamentous states, a critical virulence trait (12, 13). C. albicans was incubated with 2.5 μM CpdLC-6888 overnight before being exposed to 10% heat-inactivated fetal bovine serum (HI FBS) at 37°C for 4 h in the presence or absence of 2.5 μM CpdLC-6888. This concentration of compound was selected as it had minimal impact on growth under these conditions. While C. albicans filamented in the presence of serum alone, the addition of CpdLC-6888 blocked filamentation, akin to the noninducing condition (Fig. 1F). Therefore, our chemical screen identified a molecule with novel antifungal activity against C. albicans, which was also able to impede hyphal morphogenesis at subinhibitory concentrations.

### CpdLC-6888 putatively inhibits ergosterol biosynthesis through targeting of Erg11.

**FIG 2:**
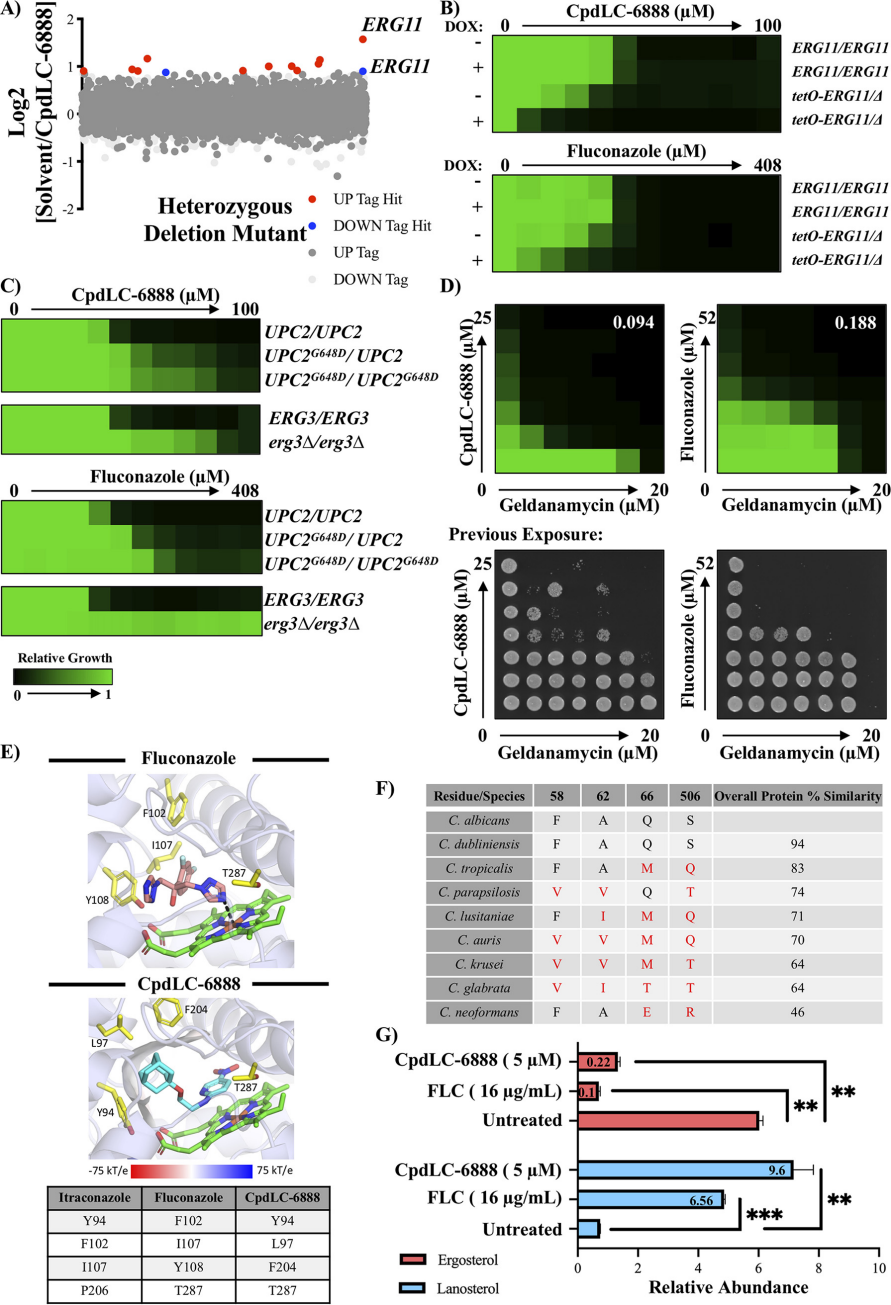
CpdLC-6888 putatively targets Erg11. (A) Haploinsufficiency profiling (HIP) was performed using a library of double-barcoded heterozygous deletion mutants grown in the presence or absence of 0.6 μM CpdLC-6888. The relative abundance of each strain was assessed by high-throughput sequencing to quantify the abundance of two strain-specific barcodes (UP-TAG and DN-TAG). Strains were considered significantly reduced in abundance if the solvent/drug log_2_ ratio was greater than 5.5 median absolute deviations (MADs) above the median for both the UP-TAG and DOWN-TAG or if either the UP-TAG or DOWN-TAG was >5.5 MAD and the opposing TAG was omitted due to low total reads. Significantly enriched UP-TAGs and DOWN-TAGs are shown in blue and red, respectively. Gray dots represent strains that were not classified as hits because neither tag was significant or only one tag was significant, but the other had reads above threshold. (B) CpdLC-6888 and fluconazole dose-response assays performed as described for Fig. 1C with a slight modification. For the parental CaSS1 (*ERG11/ERG11*) and *tetO-ERG11/erg11Δ* C. albicans strains, cultures were grown overnight in YPD followed by an additional night in YPD supplemented with 0.01 μg/mL doxycycline (DOX) to repress *ERG11* expression. Strains were then inoculated into assay plates with or without DOX, fluconazole, and CpdLC-6888 as described for Fig. 1C. See color bar. (C) CpdLC-6888 and fluconazole dose-response assays performed as described for Fig. 1C with either the parental SC5314 (*UPC2/UPC2*) and *UPC2^G648D^* mutant C. albicans strains (top) or with the parental SN95 (*ERG3/ERG3*) and *erg3Δ/erg3Δ* strains (bottom). See color bar in panel B. (D, top) Dose-response matrices with C. albicans CaCi-2 comparing CpdLC-6888 or fluconazole in combination with geldanamycin. Growth conditions and data analysis were as described for Fig. 1C. See color bar in panel B. Spotting assays (bottom) were performed by removing 2.5 μL of cells from the dose-response matrices after 48 h of growth and spotting onto drug-free YPD agar. Plates were incubated at 30°C for 24 h before being photographed. FICI_90_ values are denoted in the upper right. (E) Predicted binding poses from docking Erg11 and heme (green structure) with fluconazole (pink structure, top) and CpdLC-6888 (light blue structure, middle). Within the structures, nitrogen (dark blue), iron (orange), oxygen (red), and fluorine (pale blue) are also indicated. Interacting residues between Erg11 and the molecules are listed in the table and indicated on the structures (yellow) along with the metal coordination bond (black). The hydrophobic patch is indicated by the electrostatic surface. (F) Table listing Erg11 residues present at indicated position from each fungal species as well as overall protein similarity. (G) The abundance of ergosterol (pink) and lanosterol (blue) was quantified by LC-MS in C. albicans SN95 cells treated with solvent, CpdLC-6888, or fluconazole. Values are relative to an internal cholesterol standard, and the fold change relative to the untreated sample is indicated in each respective bar. Errors represent means ± SD from technical triplicates, and significance was determined by a Welch’s two-sided unpaired *t* test comparing untreated to each treated condition. **, *P*  < 0.01; ***, *P*  < 0.001.

To gain insights into the putative target(s) of CpdLC-6888, haploinsufficiency profiling (HIP) was performed (Text S1). HIP operates under the principle that deletion of one copy of a compound’s target gene, or genes involved in related pathways, in a diploid organism will confer hypersensitivity to the compound (14, 15). The relative growth of each C. albicans strain in a library of double-barcoded heterozygous deletion mutants covering ~90% of the genome was assessed in the presence of CpdLC-6888 compared to a solvent control (14). Strain-specific molecular barcodes were sequenced, and strains carrying barcodes with a solvent/drug log_2_ ratio of reads greater than 5.5 median absolute deviations (MADs) above the median of the entire pool were scored as significantly hypersensitive. Interestingly, the heterozygous deletion mutant for *ERG11*, the target of the azoles, emerged as the only significant hit identified from analysis of both barcodes (Fig. 2A).

Erg11 was further explored as the putative target of CpdLC-6888 by examining changes in susceptibility when *ERG11* expression was altered. First, the effect of gene repression was assessed with a tetracycline-repressible C. albicans strain in which addition of doxycycline (DOX) repressed *ERG11* expression (see Fig. S1 in the supplemental material) (16). Dose-response assays with and without DOX against CpdLC-6888 or the widely deployed azole fluconazole revealed that repression of *ERG11* conferred hypersensitivity to both CpdLC-6888 and fluconazole (Fig. 2B). Second, the effect of overexpression of *ERG11* was examined using strains with a heterozygous and homozygous gain-of-function mutation in the *ERG11* transcriptional regulator *UPC2* (Fig. S1). The mutation conferred increased levels of resistance to CpdLC-6888 in a manner similar to that of fluconazole, with the highest resistance seen in the homozygous mutant that also showed the highest expression of *ERG11* (Fig. 2C and Fig. S1).

10.1128/msphere.00075-22.1 FIG S1 Relative expression of *ERG11* in select C. albicans strains. Transcript levels of *ERG11* in the CaSS1 and *tetO-ERG11/erg11Δ* strains (blue) in the presence and absence of 0.01 μg/mL DOX and the SC5314, *UPC2/UPC2^G648D^*, and *UPC2^G648D^*/*UPC2^G648D^* strains (pink) was quantified by RT-qPCR. Transcript levels are relative to *ACT1* and *GPD1* levels, and error bars represent SEM. Significance was determined using a one-way ANOVA with the means of technical triplicates. ***, *P* < 0.001; ****, *P*  < 0.0001. Download FIG S1, TIF file, 0.4 MB. Copyright © 2022 Du Bois et al. 2022 Du Bois et al. https://creativecommons.org/licenses/by/4.0/ This content is distributed under the terms of the Creative Commons Attribution 4.0 International license.

Resistance to azoles is also achieved by loss-of-function mutations in *ERG3*, a gene that encodes an enzyme in the ergosterol biosynthesis pathway responsible for producing an aberrant sterol intermediate during azole exposure (4). There was a significant increase in resistance to both CpdLC-6888 and fluconazole upon homozygous deletion of *ERG3* (Fig. 2C), consistent with the model that CpdLC-6888 inhibits Erg11. These findings are further supported by the lack of activity of CpdLC-6888 seen in the azole-resistant clinical isolate CaCi-17 (Fig. 1D).

The results thus far support CpdLC-6888 as targeting Erg11 in an azole-like manner, despite the fact that it does not possess the canonical five-membered nitrogen-containing azole ring. A well-established feature of the azoles is their synergistic interaction with inhibitors of the molecular chaperone Hsp90, such as geldanamycin. Further, combination treatment results in fungicidal activity (17, 18). To determine if CpdLC-6888 acts in a similar manner, dose-response matrices were performed with CpdLC-6888 and geldanamycin. Compound interaction was quantified using the fractional inhibitory concentration index 90 (FICI_90_), where synergy is represented by a value lower than 0.5 (19). These results indicated that CpdLC-6888 synergizes with geldanamycin, with an FICI_90_ value of 0.094 (Fig. 2D, top). Spotting assays in which compound-treated cells were transferred onto drug-free medium were used to assess the cidality of this chemical interaction. Both fluconazole and CpdLC-6888 were found to be fungistatic alone at concentrations that inhibited growth but became fungicidal when combined with geldanamycin (Fig. 2D, bottom).

To investigate potential interactions between CpdLC-6888 and Erg11, computational docking using Rosetta was employed to model binding poses compared to fluconazole (Text S1). Docking of fluconazole returned the expected metal coordination bond between the azole ring nitrogen and heme iron (Fig. 2E, top) (20, 21). However, given the absence of the azole ring in CpdLC-6888, the same metal coordination bond was not identified by docking analysis (Fig. 2E, bottom). Instead, docking analysis implicated four candidate residues in the interaction between Erg11 and CpdLC-6888: Y94, L97, F204, and T287. CpdLC-6888 and itraconazole both interact with Y94, while CpdLC-6888 and fluconazole both interact with T287. Thus, this analysis predicts similar amino acids are required for CpdLC-6888 to bind to Erg11 compared to azoles, despite a vastly different compound structure. A close examination of the binding mode unveiled it is the adamantyl group of CpdLC-6888 that interacts with L97 and F204, which form a hydrophobic patch of the binding pocket, interactions that do not occur with azoles. Additional analysis comparing Erg11 sequence within the binding pocket residues across fungal pathogens revealed four residues that are not conserved across the protein in diverse *Candida* species (Fig. 2F). Interestingly, these residues are found at the opening of the drug-protein binding pocket based on the existing Erg11 structure, and conservation of these residues approximately correlates with the biological activity observed in Fig. 1E.

Finally, to specifically probe Erg11 inhibition, the abundances of the Erg11 precursor lanosterol and the pathway’s end product, ergosterol, were quantified through sterol profiling. C. albicans was treated with equivalent inhibitory concentrations of CpdLC-6888 or fluconazole, and sterol abundance was evaluated by LC-MS. Consistent with fluconazole, CpdLC-6888 treatment resulted in a significant increase in lanosterol and a significant decrease in ergosterol relative to solvent-treated controls (Fig. 2G). Collectively, these data provide evidence that CpdLC-6888 targets Erg11 in C. albicans. These findings are intriguing, because while the azoles are established inhibitors of Erg11, CpdLC-6888 appears to share the same target despite lacking the canonical azole ring (4, 22). This scaffold, therefore, expands the repertoire of chemical matter capable of inhibiting Erg11 (23). In the current climate where rapidly progressing antifungal resistance paired with a dearth of antifungal treatment options threatens our health care system, CpdLC-6888 represents a new scaffold to explore in an effort to expand our antifungal arsenal.

10.1128/msphere.00075-22.2 TABLE S1 Strains used in this study. Download Table S1, DOCX file, 0.03 MB. Copyright © 2022 Du Bois et al. 2022 Du Bois et al. https://creativecommons.org/licenses/by/4.0/ This content is distributed under the terms of the Creative Commons Attribution 4.0 International license.

10.1128/msphere.00075-22.3 TABLE S2 Primers used in this study. Download Table S2, DOCX file, 0.01 MB. Copyright © 2022 Du Bois et al. 2022 Du Bois et al. https://creativecommons.org/licenses/by/4.0/ This content is distributed under the terms of the Creative Commons Attribution 4.0 International license.
