# Supplementary material for: High-Throughput Chemical Screen Identifies a 2,5-Disubstituted Pyridine as an Inhibitor of Candida albicans Erg11
Source: mSphere. 2022 May 9;7(3):e00075-22. doi: 10.1128/msphere.00075-22 (PMC9241532; doi:10.1128/msphere.00075-22)
Supplement: TEXT S1 [file msphere.00075-22-s0001.docx]

**Supplementary Methods**

**Culture conditions and strains**

Strains used in this study are listed in Table S1. All fungal strains were stored in 25% glycerol in YPD medium (1% yeast extract, 2% peptone, and 2% D-glucose) and maintained at −80 °C. Strains were revived by streaking onto YPD agar plates (2% agar; Bioshop) and incubating for 24 hours at 30 °C, before storage at 4 °C for up to three weeks. Strains were always grown overnight in YPD medium prior to experimental use and subsequently grown in YPD unless otherwise indicated.

**Chemical screen of synthetic compounds**

A screen for antifungal activity against the *C. albicans* CaSS1 strain was conducted with a 9,600-compound subset of the University of Tokyo Core Chemical Library as previously described (2). 0.2 µL of each 10 mM DMSO (Sigma) compound stock was transferred from original plates to a 384-well screening plate (Corning) using a BioTech ADS-384-8 compound dispenser. Dispenser cartridges were washed with DMSO and dried between each stock plate. A *C. albicans* saturated YPD overnight culture was used to inoculate YPD medium with ~1x10^3^ cells/mL. A Rainin Liquidator™ 96 was then used to dispense 20 µL of this dilution into the wells of each plate resulting in a screening concentration of 10 µM. Plates were incubated for 24 hours at 30 °C before mixing using an Eppendorf MixMate™ (Fisher Scientific) for 15 seconds. Growth was quantified by reading optical density at 600 nm (OD_600_) with a SpectraMax M2 (Molecular Devices). The percentage growth of CaSS1 was calculated relative to the average growth in DMSO controls for each compound, and those compounds demonstrating ≥ 80% growth inhibition were ordered from an external supplier for subsequent analyses.

**Chemical susceptibility assays**

Compound potency was assessed by dose-response assays, and chemical interactions were assessed by dose-response matrices in 96-well plates as previously described (3). Wells were filled with 100 µL of YPD medium inoculated with ~1x10^3^ cells/mL of the indicated strain from a saturated overnight culture. A two-fold gradient of fluconazole (Sequoia Research Products), CpdLC-2800 (MCULE-9642413447), CpdLC-376 (MolPort-000-738-392), CpdLC-6888 (MolPort-002-731-528), or geldanamycin (Toronto Research Chemicals Inc.), as indicated, was dispensed into each well with a Tecan D300e compound dispenser. Plates were incubated at 30 °C for the indicated time. A spectrophotometer (Molecular Devices SpectraMax Plus 384) was used to quantify growth by measuring OD_600_. All assays were performed in biological and technical duplicate. Values were corrected for medium background, and growth was normalized to respective untreated control wells and plotted using Heatmapper (4). For dose-response matrices, fractional inhibitory concentration index at 90% growth inhibition (FICI_90_) was calculated using the formula (5):

$FICI_{90}=\left( \frac{MIC_{Drug A Combo}}{MIC_{Drug A Alone}}+\frac{MIC_{Drug B Combo}}{MIC_{Drug B Alone}} \right)$

For genetic depletion of *ERG11* with the *tetO-ERG11/erg11∆* strain, saturated overnight cultures of the *tetO-ERG11/erg11∆* strain and CaSS1 parent were diluted 1:100 into fresh YPD medium. 0.01 µg/mL of doxycycline (DOX) was added to the drug-treated samples and tubes were incubated overnight at 30 °C with shaking. Cells were then used for dose response assays as described above, but with 0.01 µg/mL DOX supplementing the indicated inoculum stocks.

For kinetic growth assays, the parental *C. albicans* CaSS1 strain along with heterozygous mutants identified as hits from the HIP analysis (Table S1), were assessed for hypersensitivity to CpdLC-6888. YPD medium was inoculated with ~1x10^3^ cells/mL and 40 µL was dispensed into a 384-well microtiter plate. Triplicate wells for each strain were left untreated or treated with 0.8 µM CpdLC-6888. Plates were grown in static conditions at 30 °C and OD_600_ was measured every 15 minutes for 24 hours using a growth curve robot (S&P Robotics Inc.). Data were analyzed in Microsoft Excel (Version 16.54). Relative growth of heterozygous deletion strains was determined by calculating the ratio of the area under the curve (AUC) in the presence of 0.8 µM CpdLC-6888 relative to the AUC in drug-free YPD medium. Strains were all assessed in biological duplicate and technical triplicate.

**Cidality assays**

Cells previously exposed to compounds for 48 hours in dose-response matrices were spotted for cidality. 2.5 µL was extracted from each well with a multi-channel pipette and dispensed onto drug-free YPD agar plates. Plates were incubated at 30 °C for 24 hours and photographed using a ChemiDoc Imaging System (BioRad). All experiments were performed in biological and technical duplicate.

**Filamentation assay**

To assess the effect of CpdLC-6888 on filamentation, we performed the protocol as previously described. (6) In brief, *C. albicans* CaSS1 was transferred from a culture grown overnight in a 96-well plate to a fresh plate with 100 µL of YPD alone or containing 2.5 µM of CpdLC-6888 using a 96-well replicator. The plate was incubated overnight at 30 °C or 37 °C. This culture was then pinned into 100 μL of fresh YPD with or without 10% heat-inactivated fetal bovine serum (HI FBS; Gibco) in the absence or presence of 2.5 µM CpdLC-6888 and plates were incubated for four hours at either 30 °C or 37 °C, consistent with the previous overnight. Cells were imaged at 40X magnification using a Zeiss Axio Imager.MI (Carl Zeiss). Experiment was conducted in biological and technical duplicate.

**Haploinsufficiency profiling (HIP)**

Pooled heterozygous (HET) double-barcoded deletion mutant (7) stocks in 25% glycerol (stored at -80 °C) were thawed and diluted to an OD_600_ of 0.1 in triplicate 60 mL YPD cultures. These were grown for 90 minutes in shaking conditions at 30 °C before being diluted two-fold in 5 mL YPD in the presence or absence of 0.6 µM CpdLC-6888. Cultures were grown for 18 hours in shaking conditions at 30 °C before harvesting cells through centrifugation. Cell pellets were stored at -80 °C. Genomic DNA was then extracted using a PureLink Genomic DNA Mini Kit (Invitrogen) and barcodes were amplified from 56 ng of genomic DNA using PCR. UP-TAG and DN-TAG pools were combined in equal quantities and sequenced using an Illumina NextSeq500 instrument (Mid-Output, V2 Chemistry). Amplification and sequencing primers used are included in Table S2. Read frequencies for both tags in each indexed sample were compiled for each strain. Those strains for which either the UP-TAG or DN-TAG in the solvent control was <20% of the median read frequency were excluded from further analysis. Log2 fold changes for the UP-TAG and DN-TAG of each strain were calculated. A strain was considered to be significantly reduced in abundance if the log2 fold change ratio of solvent:drug was > 6 median absolute deviations (MADs) above the overall median in both the UP-TAG and DOWN-TAG, or if one of the UP-TAG and DOWN-TAG was >6MAD and the opposing TAG was omitted due to failure to amplify. (UP-TAG 6MAD = 0.877, DOWN-TAG 6MAD=0.804).

**Computational Docking**

To generate potential ligand binding modes, Rosetta was used with flexible XML protocols to simultaneously dock the molecule of interest with the heme cofactor (8, 9). Molecules were docked into the binding pocket of Erg11 using an existing structure (PDB: 5V5Z). The ligand and cofactor models were downloaded from the PubChem database (10). The conformer ligand libraries were generated using BCL::Conformer (11). The protein structure was minimized using Rosetta Relax. The Rosetta docking protocol incorporates ligand and sidechain flexibility, random perturbation of the ligands through rotation and translation, and optimization through Monte Carlo minimization. 500 docking trials were completed for each molecule and the top 200 models were filtered based on the Rosetta Total Score and were further clustered and sorted based on the ligand interface energy. The model with the highest interface energy was selected for reference. Figures of the docking poses were generated with PyMOL (12). The Protein-Ligand Interaction Profiler was used to characterize the receptor interactions (13).

**Extraction and quantification of sterols**

To quantify the abundance of sterols in *C. albicans,* targeted metabolomics profiling was used as described previously (14, 15)*.* *C. albicans* (SN95) saturated overnight cultures were diluted to an OD_600_ of 0.1 in 10 mL of minimal medium (0.17% yeast nitrogen base without amino acids, without ammonium sulfate, 2% D-glucose, 0.1% monosodium glutamate, 1X histidine, 1X arginine) in the presence or absence of the indicated compound concentration for 18 hours at 30 °C with agitation. Cells were then pelleted and washed with 1X PBS. OD_600_ was measured and used to normalize cultures at which point pellets were resuspended to a final volume of 100 µL 1X PBS. Cell suspension was treated with 1 mL methanol/CHCl3 (2:1 v/v) supplemented with 0.01% w/v butylated hydroxytoluene before adding acid washed glass beads to each sample and vortexing for 10 minutes. Samples were pelleted by centrifugation for at 16,000 ×g for five minutes. The supernatant was transferred to a new vial. 400 μL of 50 mM citric acid in H_2_O, and 600 μL CHCl_3_ was added and vials were vortexed for 10 minutes before centrifuging at 16,000 ×g for five minutes. The organic phase was collected and dried in borosilicate glass tubes and dried by Genevac (EZ-2 Series SP Scientific).

For LC-MS analysis, samples were resuspended in 100 µL ethanol with an internal cholesterol standard (50 µM) included. The system used was an Acquity UPLC I-Class coupled to a Xevo G2-S QToF (Waters) and sample separation was done on an Acquity UPLC BEH C18 column (1.7 μm, 2.1 × 50 mm). Samples were separated with a gradient of 70% B to 98% B over 2.5 minutes and held at 98% B until 12 minutes at 0.2 mL/minute (A: water + 0.1% (v/w) formic acid, B: acetonitrile + 0.1 (v/v) formic acid). An APCI corona needle and electrospray ionization (ESI) were used with ESCi multi-mode ionization in positive ion mode. Selective reaction monitoring was used to detect sterols and TargetLynx (Waters; version 4.1) was used for quantification, peak finding, smoothing, and area calculations. The mass transitions were ergosterol 396.339 🡪 363.305 and 396.339 🡪 337.289, lanosterol 408.375 🡪 393.339, and cholesterol 369.35 🡪 147.1114 and 369.35 🡪 161.1274. Experiment performed in five biological replicates and technical triplicate for each sample. Data analyzed with Microsoft Excel (Version 16.54).

**Real-time quantitative PCR (RT-qPCR)**

In order to assess transcriptional changes in *ERG11* expression, the *C. albicans* CaSS1, *tetO-ERG11/erg11∆,* SC5314, *UPC2^G648D^/UPC2* and *UPC2^G648D^/ UPC2^G648D^* strains were grown overnight in YPD at 30 °C in shaking conditions. The overnights were subsequently diluted 1:100 into fresh YPD. The CaSS1 and *tetO-ERG11/erg11∆* strains were subcultured in the absence or presence of 0.01 µg/mL DOX. Cultures were incubated overnight again in the same conditions. Cells were pelleted by centrifugation at 700 xg for one minute at 4 °C, washed with cold water, and pelleted by centrifugation at 15339 xg for ~15 seconds, before supernatant was removed and pellets were flash-frozen in liquid nitrogen and stored at -80 °C.

RNA was isolated from pellets using a QIAGEN RNeasy Mini Kit and subjected to DNase treatment using an Invitrogen™ DNA-free™ DNA Removal Kit. Complementary DNA was synthesized using an iScript™ cDNA Synthesis Kit (BioRad) according to the manufacturer’s instructions.

RT-qPCR was carried out using a FastSYBR Green master Mix (Applied Biosystems) and the primer pairs oLC2285/oLC2286, oLC752/oLC753, and oLC1131/oLC1132 were used for the targets *ACT1*, *GPD1*, and *ERG11* respectively (Table S2). BioRad CFX384 Real Time System was used to carry out the reaction under the following conditions: 95 °C for 3 minutes, then 95 °C for 10 seconds and 60 °C for 30 seconds for 40 cycles. Data were analyzed using BioRad CFX Manager 3.1 and all data was normalized to housekeeping genes. Data was analyzed and significance was calculated using GraphPad Prism v9. All experiments were performed in biological duplicate and technical triplicate.

**References**

1. Homann OR, Dea J, Noble SM, Johnson AD. 2009. A phenotypic profile of the *Candida albicans* regulatory network. PLoS Genet 5:e1000783.

2. Fu C, Zhang X, Veri AO, Iyer KR, Lash E, Xue A, Yan H, Revie NM, Wong C, Lin Z-Y, Polvi EJ, Liston SD, VanderSluis B, Hou J, Yashiroda Y, Gingras A-C, Boone C, O’Meara TR, O’Meara MJ, Noble S, Robbins N, Myers CL, Cowen LE. 2021. Leveraging machine learning essentiality predictions and chemogenomic interactions to identify antifungal targets. Nat Commun 12:6497.

3. Xie J, Singh-Babak S, Cowen L. 2012. Minimum inhibitory concentration (MIC) assay for antifungal drugs. Bio Protoc 2:e252.

4. Babicki S, Arndt D, Marcu A, Liang Y, Grant JR, Maciejewski A, Wishart DS. 2016. Heatmapper: web-enabled heat mapping for all. Nucleic Acids Res 44:W147–W153.

5. Odds FC. 2003. Synergy, antagonism, and what the chequerboard puts between them. J Antimicrob Chemother 52:1.

6. O’Meara TR, Veri AO, Ketela T, Jiang B, Roemer T, Cowen LE. 2015. Global analysis of fungal morphology exposes mechanisms of host cell escape. Nat Commun 6:6741.

7. Xu D, Jiang B, Ketela T, Lemieux S, Veillette K, Martel N, Davison J, Sillaots S, Trosok S, Bachewich C, Bussey H, Youngman P, Roemer T. 2007. Genome-wide fitness test and mechanism-of-action studies of inhibitory compounds in *Candida albicans*. PLoS Pathog 3:e92.

8. Fleishman SJ, Leaver-Fay A, Corn JE, Strauch E-M, Khare SD, Koga N, Ashworth J, Murphy P, Richter F, Lemmon G, Meiler J, Baker D. 2011. RosettaScripts: A scripting language interface to the rosetta macromolecular modeling suite. PLoS ONE 6:e20161.

9. Lemmon G, Meiler J. 2012. RosettaLigand docking with flexible XML protocols. Methods Mol Biol 819:143–155.

10. Kim S, Chen J, Cheng T, Gindulyte A, He J, He S, Li Q, Shoemaker BA, Thiessen PA, Yu B, Zaslavsky L, Zhang J, Bolton EE. 2020. PubChem in 2021: new data content and improved web interfaces. Nucleic Acids Res 49:D1388–D1395.

11. Kothiwale S, Mendenhall JL, Meiler J. 2015. BCL::Conf: small molecule conformational sampling using a knowledge based rotamer library. J Cheminform 7:47.

12. Schrödinger, LLC. 2015. The PyMOL Molecular Graphics System, Version 1.8.

13. Adasme MF, Linnemann KL, Bolz SN, Kaiser F, Salentin S, Haupt VJ, Schroeder M. 2021. PLIP 2021: expanding the scope of the protein–ligand interaction profiler to DNA and RNA. Nucleic Acids Research 49:W530–W534.

14. Hoepfner D, Karkare S, Helliwell SB, Pfeifer M, Trunzer M, De Bonnechose S, Zimmerlin A, Tao J, Richie D, Hofmann A, Reinker S, Frederiksen M, Movva NR, Porter JA, Ryder NS, Parker CN. 2012. An integrated approach for identification and target validation of antifungal compounds active against Erg11p. Antimicrob Agents Chemother 56:4233–4240.

15. Iyer KR, Camara K, Daniel-Ivad M, Trilles R, Pimentel-Elardo SM, Fossen JL, Marchillo K, Liu Z, Singh S, Muñoz JF, Kim SH, Porco JA, Cuomo CA, Williams NS, Ibrahim AS, Edwards JE, Andes DR, Nodwell JR, Brown LE, Whitesell L, Robbins N, Cowen LE. 2020. An oxindole efflux inhibitor potentiates azoles and impairs virulence in the fungal pathogen Candida auris. Nat Commun 11:6429.
