# Supplementary figures and images for: High-Throughput Chemical Screen Identifies a 2,5-Disubstituted Pyridine as an Inhibitor of Candida albicans Erg11
Source: mSphere. 2022 May 9;7(3):e00075-22. doi: 10.1128/msphere.00075-22 (PMC9241532; doi:10.1128/msphere.00075-22)

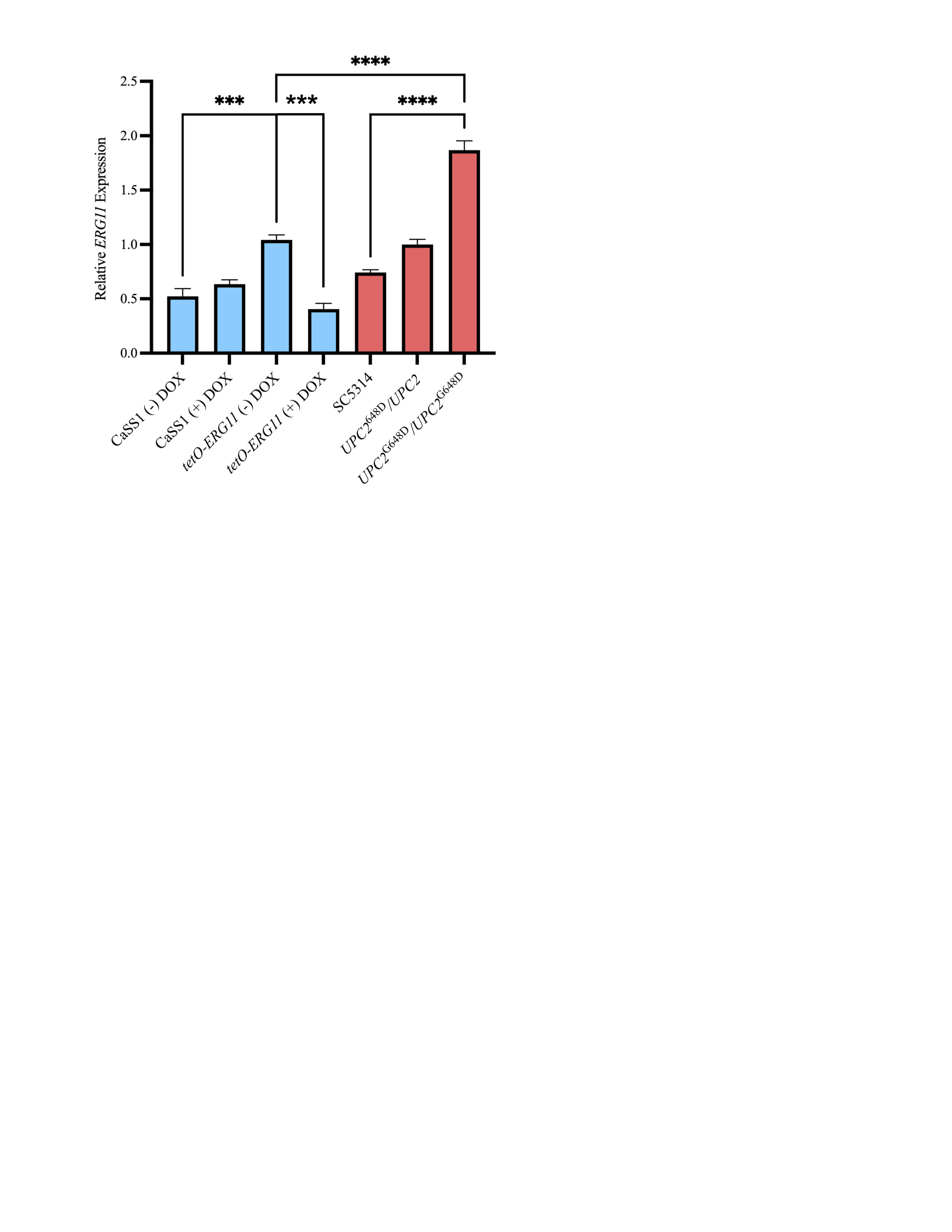

Supplement: FIG S1 [file msphere.00075-22-sf001.tif]
