# Supplementary material for: High-Throughput Chemical Screen Identifies a 2,5-Disubstituted Pyridine as an Inhibitor of Candida albicans Erg11
Source: mSphere. 2022 May 9;7(3):e00075-22. doi: 10.1128/msphere.00075-22 (PMC9241532; doi:10.1128/msphere.00075-22)
Supplement: TABLE S1 [file msphere.00075-22-st001.docx]

**Table S1: Strains used in this study**

| **Strain Name** | **Alias** | **Genotype** | **Source** |
| --- | --- | --- | --- |
| CdLC743 | *Candida dubliniensis* 36 (CD36) | Clinical isolate | (1) |
| CtLC575 | *Candida tropicalis* 1685 | Clinical isolate | Heitman Lab |
| CpLC6144 | *Candida parapsilosis* (ATCC® 22019™) | Clinical isolate | ATCC |
| ClLC572 | *Candida lusitaniae* (ATCC® 42720™) | Clinical isolate | ATCC |
| CauLC5294 | *Candida auris* 0387 (B8441) | Clinical isolate Clade I | (2)(3) |
| CkLC571 | *Candida krusei* 1680 | Clinical isolate | Heitman Lab |
| CgLC1002 | *Candida glabrata* BG2 | Clinical isolate | (4) |
| CnLC3142 | *Cryptococcus neoformans* H99 | Clinical isolate | (5) |
| CaLC155 | *Candida albicans* SC5314 | Wild-type | (6) |
| CaLC6106 | *Candida albicans* GRACE parent strain (CaSS1) | *ura3∷imm^434^/ura∷imm^434^*  *his3∷hisG/his3∷hisG*  *leu2∷tetR-GAL4AD-URA/LEU2* | (7) |
| CaLC239 | *Candida albicans* SN95 | *arg/arg4 his1/his1 URA3/ura3::imm434 IRO1/iro1::imm434* | (8) |
| CaLC79 | *Candida albicans* CaCi-2 | Clinical isolate | (9) |
| CaLC91 | *Candida albicans* CaCi-17 | Clinical isolate | (9) |
| CaLC1764 | F5 | Clinical isolate | (10) |
| CaLC1767 | G5 | Clinical isolate | (10) |
| CaLC5544 | *TAC1/tac1Δ* | *arg4 /arg4 , leu2 /leu2 ::LEU2(Ca), his1 /his1 ::HIS1(Ca), URA3/ura3 , IRO1/iro1, tac1::HIS::TAC1WT-SAT1/tac1::LEU* | (11) |
| CaLC5547 | *TAC1^M677Δ^ /tac1Δ* | As CaLC5544, *tac1::HIS:: TAC1^M677Δ^-SAT1/tac1::LEU* | (11) |
| CaLC5550 | *TAC1^N972D^/tac1Δ* | As CaLC5544, *tac1::HIS:: TAC1^N972D^-SAT1/tac1::LEU* | (11) |
| CaLC5553 | *TAC1^N977D^/tac1Δ* | As CaLC5544, *tac1::HIS:: TAC1 ^N977D^ -SAT1/tac1::LEU* | (11) |
| CaLC990 | *Candida albicans* DPL15 | Clinical isolate, *FKS1^F641S^* | (12) |
| CaLC1775 | *Candida albicans* SCUPC2R12A | As CaLC155, *UPC2^G648D^-FRT/UPC2-2* | (13) |
| CaLC1776 | *Candida albicans* SCUPC2R14A | As CaLC155, *UPC2^G648D^-FRT/UPC2^G648D^-FRT* | (13) |
| CaLC4097 | *Candida albicans tetO-ERG11/erg11∆* | As CaLC6106, *SAT1∷tetO-ERG11/erg11∷HIS3* | (7) |
| CaLC660 | *Candida albicans erg3∆/erg3∆* | *arg4 /arg4 his1 /his1 URA3/ura3::imm434 IRO1/iro1::imm434 CaTAR::HIS3 erg3::FRT/erg3::FRT* | (14) |

**References**

1. Sullivan DJ, Westerneng TJ, Haynes KA, Bennett DE, Coleman DC. 1995. *Candida dubliniensis* sp. nov.: phenotypic and molecular characterization of a novel species associated with oral candidosis in HIV-infected individuals. Microbiology 141:1507–1521.

2. Lockhart SR, Etienne KA, Vallabhaneni S, Farooqi J, Chowdhary A, Govender NP, Colombo AL, Calvo B, Cuomo CA, Desjardins CA, Berkow EL, Castanheira M, Magobo RE, Jabeen K, Asghar RJ, Meis JF, Jackson B, Chiller T, Litvintseva AP. 2017. Simultaneous emergence of multidrug-resistant *Candida auris* on 3 continents confirmed by whole-genome sequencing and epidemiological analyses. Clin Infect Dis 64:134–140.

3. Lutgring JD, Machado M-J, Benahmed FH, Conville P, Shawar RM, Patel J, Brown AC. 2018. FDA-CDC antimicrobial resistance isolate bank: a publicly available resource to support research, development, and regulatory requirements. J Clin Microbiol 56:e01415-17.

4. Cormack BP, Falkow S. 1999. Efficient homologous and illegitimate recombination in the opportunistic yeast pathogen *Candida glabrata*. Genetics 151:979–987.

5. Granger DL, Perfect JR, Durack DT. 1985. Virulence of *Cryptococcus neoformans*. Regulation of capsule synthesis by carbon dioxide. J Clin Invest 76:508–516.

6. Gillum AM, Tsay EYH, Kirsch DR. 1984. Isolation of the *Candida albicans* gene for orotidine-5′-phosphate decarboxylase by complementation of *S. cerevisiae ura3* and *E. coli pyrF* mutations. Molec Gen Genet 198:179–182.

7. Roemer T, Jiang B, Davison J, Ketela T, Veillette K, Breton A, Tandia F, Linteau A, Sillaots S, Marta C, Martel N, Veronneau S, Lemieux S, Kauffman S, Becker J, Storms R, Boone C, Bussey H. 2003. Large-scale essential gene identification in *Candida albicans* and applications to antifungal drug discovery: *C. albicans* essential gene identification and antifungal drug discovery. Mol Microbiol 50:167–181.

8. Noble SM, Johnson AD. 2005. Strains and strategies for large-scale gene deletion studies of the diploid human fungal pathogen *Candida albicans*. Eukaryot Cell 4:298–309.

9. White TC. 1997. The presence of an R467K amino acid substitution and loss of allelic variation correlate with an azole-resistant lanosterol 14⍺ demethylase in *Candida albicans*. Antimicrob Agents Chemother 41:1488–1494.

10. Dunkel N, Blaß J, Rogers PD, Morschhäuser J. 2008. Mutations in the multi-drug resistance regulator *MRR1*, followed by loss of heterozygosity, are the main cause of *MDR1* overexpression in fluconazole-resistant *Candida albicans* strains. Mol Microbiol 69:827–840.

11. Iyer KR, Camara K, Daniel-Ivad M, Trilles R, Pimentel-Elardo SM, Fossen JL, Marchillo K, Liu Z, Singh S, Muñoz JF, Kim SH, Porco JA, Cuomo CA, Williams NS, Ibrahim AS, Edwards JE, Andes DR, Nodwell JR, Brown LE, Whitesell L, Robbins N, Cowen LE. 2020. An oxindole efflux inhibitor potentiates azoles and impairs virulence in the fungal pathogen *Candida auris*. Nat Commun 11:6429.

12. Singh SD, Robbins N, Zaas AK, Schell WA, Perfect JR, Cowen LE. 2009. Hsp90 governs echinocandin resistance in the pathogenic yeast *Candida albicans* via calcineurin. PLoS Pathog 5:e1000532.

13. Heilmann CJ, Schneider S, Barker KS, Rogers PD, Morschhäuser J. 2010. An A643T mutation in the transcription factor Upc2p causes constitutive *ERG11* upregulation and increased fluconazole resistance in *Candida albicans*. Antimicrob Agents Chemother 54:353–359.

14. Robbins N, Collins C, Morhayim J, Cowen LE. 2010. Metabolic control of antifungal drug resistance. Fungal Genetics and Biology 47:81–93.
