# Supplementary material for: High-Throughput Chemical Screen Identifies a 2,5-Disubstituted Pyridine as an Inhibitor of Candida albicans Erg11
Source: mSphere. 2022 May 9;7(3):e00075-22. doi: 10.1128/msphere.00075-22 (PMC9241532; doi:10.1128/msphere.00075-22)
Supplement: TABLE S2 [file msphere.00075-22-st002.docx]

**Table S2: Primers used in this study.**

| Primer Description | Sequence (5’ to 3’) |
| --- | --- |
| HIP UP-TAG universal amplification primer | AATGATACGGCGACCACCGAGATCTACACCGAG  GTCGAGAATGATGTCCACGAGGTCTCT |
| HIP UP-TAG index amplification primer (1) | CAAGCAGAAGACGGCATACGAGATNNNNNNGCC  ATTTGTCTGTCGACCTGCAGCGTACG |
| HIP DOWN-TAG universal amplification primer | AATGATACGGCGACCACCGAGATCTACACCACAT  GATATGTTGAGCGGTGTCGGTCTCGTAG |
| HIP DOWN-TAG index amplification primer (1) | CAAGCAGAAGACGGCATACGAGATNNNNNNGAGT  ATCTGTATCTGGCC GAGCTCGAATTCATCGAT |
| HIP UP-TAG sequencing primer | CGAGGTCGAGAATGATGTCCACGAGGTCTCT |
| HIP DOWN-TAG sequencing primer | CACATGATATGTTGAGCGGTGTCGGTCTCGTAG |
| HIP UP-TAG index sequencing primer | CGTACGCTGCAGGTCGACAGACAAATGGC |
| HIP DOWN-TAG index sequencing primer | ATCGATGAATTCGAGCTCGGCCAGATACAGATACTC |
| oLC752-*GPD1*-Forward | AGTATGTGGAGCTTTACTGGGA |
| oLC753-*GPD1*-Reverse | CAGAAACACCAGCAACATCTTC |
| oLC1131-*ERG11*-Forward | GATGTTTCTGCTGAAGATGC |
| oLC1132-*ERG11*-Reverse | ATAGTTGAGCAAATGAACGG |
| oLC2285-*ACT1*-Forward | GACCTTGAGATACCCAATTG |
| oLC2286-*ACT1­-*Reverse | CAGCTTGAATGGAAACGTAG |

* For solvent triplicates NNNNNN= CACGAT, CACTCA, CAGGCG and for CpdLC-6888 treatment triplicates NNNNNN= CATGGC, CATTTT, and CCAACA.
